# Light perception in aerial tissues enhances DWF4 accumulation in root tips and induces root growth

**DOI:** 10.1038/s41598-017-01872-4

**Published:** 2017-05-12

**Authors:** Jun Sakaguchi, Yuichiro Watanabe

**Affiliations:** Department of Life Sciences, Graduate School of Arts and Sciences, University of Tokyo, Komaba, Meguro-ku, Tokyo 153-8902 Japan

## Abstract

Many attempts have been made to characterize the activities of brassinosteroids (BRs), which are important plant hormones. The crosstalk between light perception and the BR signalling pathway has been extensively studied regarding its effects on photomorphogenesis, especially in elongating etiolated hypocotyls. In contrast, how and where the light induces BR biosynthesis remain uncharacterized. DWF4 is one of the main enzymes involved in the BR biosynthesis pathway in *Arabidopsis thaliana*. We established *DWF4-GUS A*. *thaliana* lines in a homozygous *dwf4-102* genetic background, but functionally complemented with a genomic *DWF4* sequence fused in-frame with a β-glucuronidase (GUS) marker gene. The *DWF4-GUS* plants enabled the visualization of the accumulation of DWF4 under different conditions. We investigated the effects of aboveground light on root and hypocotyl growth. We observed that root length increased when shoots were maintained under light irrespective of whether roots were exposed to light. We also determined that light perception in aerial tissues enhanced DWF4 accumulation in the root tips. Overall, our data indicate that BR biosynthesis is promoted in the root tip regions by an unknown mechanism in distantly located shoot tissues exposed to light, leading to increased root growth.

## Introduction

Brassinosteroids (BRs) are phytohormones commonly found in higher plants. They induce diverse responses, including cell division, cell elongation, and tissue differentiation^1–6^. Components involved in BR biosynthesis, signal perception, and cytosolic signal cascades have been determined through studies of many loss-of-function mutants or mutants insensitive to applications of exogenous BRs. Such BR-defective mutants often exhibit severe dwarfism, with the production of small leaves and short flower stems under light conditions, and stunted hypocotyls even in darkness. Additionally, mutants defective in BR biosynthesis are easily distinguished by the suppression of the dwarf phenotype following the application of exogenous BRs (*DWF1*/*CBB1* ^7–9^, *CPD*/*DWF3*/*CBB3* ^7, 10, 11^, *DWF4* ^12, 13^, *DWF5* ^14^, *DET2*/*DWF6* ^15–17^, and *DWF7*/*STE1* ^18^). In contrast, mutants in which the application of exogenous BRs failed to suppress mutant phenotypes or those that exhibited decreased sensitivity to BR biosynthesis inhibitors have provided clues regarding the genes involved in the perception of BRs at the plasma membrane (*BRI1*/*DWF2*/*CBB2* ^7, 19, 20^). These mutants also provided information about downstream cytoplasmic phosphorylation cascades (*BIN2* ^21–23^, *BSKs* ^24^, *BSU1* ^25^, and *BSS1* ^26^) and transcription factors (*BES1* ^27, 28^ and *BZR1* ^29, 30^). In addition to the BR receptor complex, co-receptors and inhibitory regulators have been identified (BAK1^31–34^ and BKI1^35–37^).

Previous studies analysed the crosstalk between light and BR signalling pathways^38, 39^. Many of the findings of these BR-related studies focused on hypocotyl growth in darkness. In contrast, BR-related mutants exhibit stunted growth in darkness. Dominant positive mutations of transcription factor genes located downstream of the BR signalling pathway result in hypocotyl elongation even in the presence of BRZ, which is an inhibitor of BR biosynthesis, or in *bri1* mutants^27, 29^. It was assumed that light conditions influence BR biosynthesis/accumulation in plants and that BRs promote, for instance, hypocotyl elongation depending on their abundance. Eventually, hypocotyl elongation in darkness became widely used as a marker of BR-induced responses^10, 13, 15^. It is assumed that during the elongation of hypocotyls, BRs are synthesized in hypocotyls and induce localized rapid cell elongation^40, 41^. The long-distance transport of BRs has not been considered.

The abovementioned analyses were conducted using aerial plant tissues. Relatively few studies have focused on the root tissues of plants growing in the shade or in darkness^42–44^. In this study, we investigated the effects of light conditions on root growth, in parallel with hypocotyl growth used as a control. We observed that roots grew more extensively when the shoots were maintained under light, regardless of whether roots were exposed to light or darkness.

We established *DWF4-GUS A*. *thaliana* lines in a *dwf4-102* homozygous genetic background. These lines were functionally complemented with a genomic *DWF4* sequence fused in-frame with a β-glucuronidase (GUS) marker gene. The *DWF4-GUS* plants enabled us to visualize the accumulation of DWF4 under various conditions. Our experiments involving *DWF4-GUS* plants revealed that exposure to light stimulates aerial tissues to induce the expression of *DWF4*, which encodes a BR biosynthesis enzyme, in root tips located distally to the illuminated tissues. Our findings strongly suggest that BR biosynthesis is promoted in roots by an unknown mechanism occurring in distantly located shoot tissues, and that the resulting BRs induce root growth.

## Results

### Establishment of plants producing the DWF4 -GUS fusion protein

**Figure 1:**
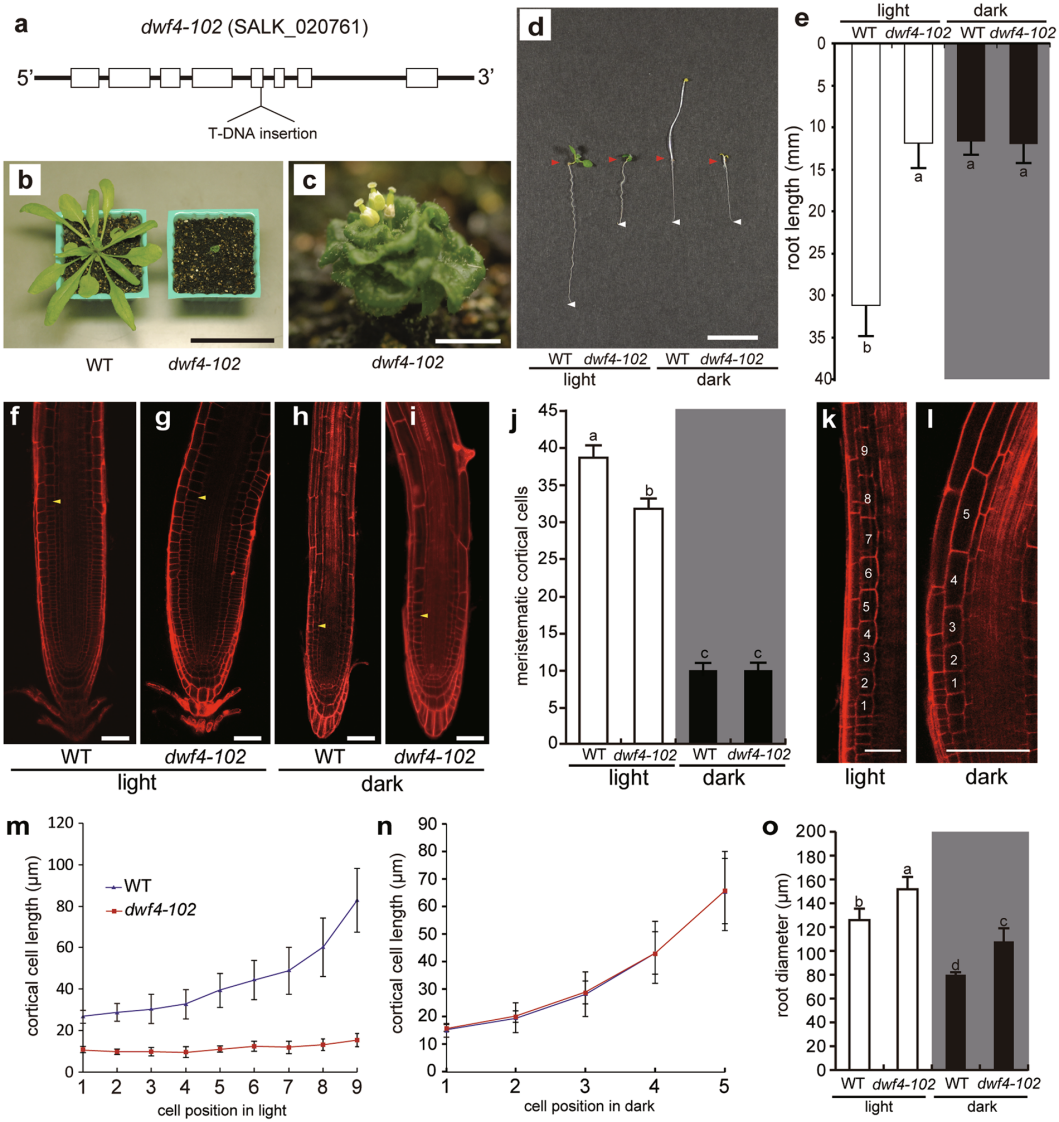
*dwf4-102* mutants. (**a**) The *dwf4-102* mutant plants contained a T-DNA insertion in the fifth exon of the *DWF4* gene. These plants exhibited dwarfism during the vegetative stage (**b**) and produced defective flowers during the reproductive stage (**c**). The roots of *dwf4-102* plants were relatively short under light and dark conditions (**d**,**e**) [wild-type (WT): n = 48; *dwf4-102*: n = 16]. Samples were stained with propidium iodine for analyses of the root apexes of WT (**f**,**h**) and *dwf4-102* mutants (**g**,**i**) incubated under light (**f**,**g**) or in darkness (**h**,**i**). Arrowheads indicate the first cell of the elongation zone. Root diameters were measured in the region corresponding to the first cell of the elongation zone. (**j**) Cell division activity in the root apical meristems. The cortical cells were counted. There were no differences between WT and *dwf4-102* plants incubated in darkness [WT: n = 42; *dwf4-102*: n = 24]. (**k**–**n**) Cell elongation activity in root tips. Under light conditions, cell elongation was observed in WT plants but not in *dwf4-102* plants (**k**,**m**) [WT: n = 16; *dwf4-102* n = 12]. On the other hand, the number of cells in the elongation zone was reduced, and there were no differences between WT and *dwf4-102* plants incubated in darkness (**l**,**n**) [WT: n = 16; *dwf4-102*: n = 12]. (**o**) Root diameter. The roots of *dwf4-102* plants were thicker than those of the WT plants under light and dark conditions [WT: n = 40; *dwf4-102*: n = 15]. Means denoted by the same letter are not significantly different (P < 0.05) according to the Tukey–Kramer method (**j**,**o**). Scale bars represent 50 mm (**b**), 10 mm (**c**,**d**), and 50 µm (**f**–**i**,**k**,**l**). Error bars represent standard deviations.

The BR biosynthesis pathway has been characterized in detail. Among the genes corresponding to the associated enzymes, we focused on *DWF4*. This gene encodes a C-22 hydroxylase, which is a key enzyme in the BR biosynthesis pathway^13, 45–48^. Among several alleles, *dwf4-102* is the most frequently used for characterizing the *dwf4* mutant^49^. It is a null allele that harbours a T-DNA insertion in the fifth exon (Fig. 1a). The *dwf4-102* mutant plants exhibit severe dwarfism due to a BR-deficiency as is often observed for other BR-related mutants^7–11, 14–23^. The mutants produce very small leaves during the vegetative stage (Fig. 1b), and there is a lack of petal expansion, stamen elongation, and stem elongation in the flowers generated during the reproductive stage (Fig. 1c). Wild-type (WT) plants grown in darkness have elongated hypocotyls, while *dwf4-102* mutant plants do not, which is common among BR mutants^12, 13^.

In addition to shorter stems, the roots of *dwf4-102* plants exposed to light are considerably shorter than those of WT plants (Fig. 1d,e). In darkness, the roots of WT and *dwf4-102* plants are similarly short. These results suggest that light positively affects root growth in WT plants, but not in *dwf4-102* plants. To clarify the reasons for the differential root growth under light conditions, an examination of cell division and elongation is required.

We first analysed root cells using confocal laser scanning microscopy after staining samples with propidium iodine (PI) (Fig. 1f–i). We examined cell division activity in root apical meristems (RAMs) to determine whether it influences root length. We counted the number of cortical cells as described by Silva-Navas *et al*.^44^. There were fewer meristematic cells in the root meristems of WT plants grown in darkness than in plants exposed to light (Fig. 1j). This result suggests that cell division was less active in darkness. The meristems in darkness had about one-fourth as many cells as those grown under light. Exposure to light increased the number of meristematic cortical cells also in *dwf4-102* plants (Fig. 1j).

We then measured cell lengths of elongating cells in elongation zone (nine cells in light condition (Fig. 1k,m) and five cells in darkness (Fig. 1l,n)) to examine whether cell elongation activity influences root length. Under light condition, the elongation of cortical cells in *dwf4-102* plants was remarkably reduced (Fig. 1m), however there were almost no difference between WT plants and *dwf4-102* plants in darkness (Fig. 1n). Interestingly, the cortical cell elongation in darkness must have been promoted by BR independent pathway. Thus, reduced cell elongation also contributed to the development of short roots in darkness. These results suggest that light enhanced root growth in WT plants by promoting cell division and elongation. It is likely that this enhanced growth is related to BR biosynthesis because it was not observed in the *dwf4-102* mutants. The roots of the *dwf4-102* mutant plants were thicker than those of the WT plants, regardless of the presence of light (Fig. 1f–i,o).

We hypothesized that light enhances *DWF4* expression and the subsequent root growth. To test the idea that light induces root-specific *DWF4* expression, we attempted to establish complementation lines that express a chimeric gene consisting of the *DWF4* genomic sequence (with its promoter and coding sequence) and the GUS gene sequence produced by translational fusion (Supplementary Fig. S1(a)). We selected three independent *DWF4-GUS* homozygous lines with a *dwf4-102* homozygous background from the T_2_ generation (i.e., *DWF4-GUS* plants #1, #12, and #17). Plants from each line exhibited normal cotyledon development and root elongation during the juvenile phase (Supplementary Fig. S1(b)), and rosette leaf development during the vegetative phase (Supplementary Fig. S1(c)). Additionally, these plants underwent bolting and developed normal fertile flowers almost simultaneously with WT plants.

### *DWF4-GUS* expression in shoots and roots under light and dark conditions

**Figure 2:**
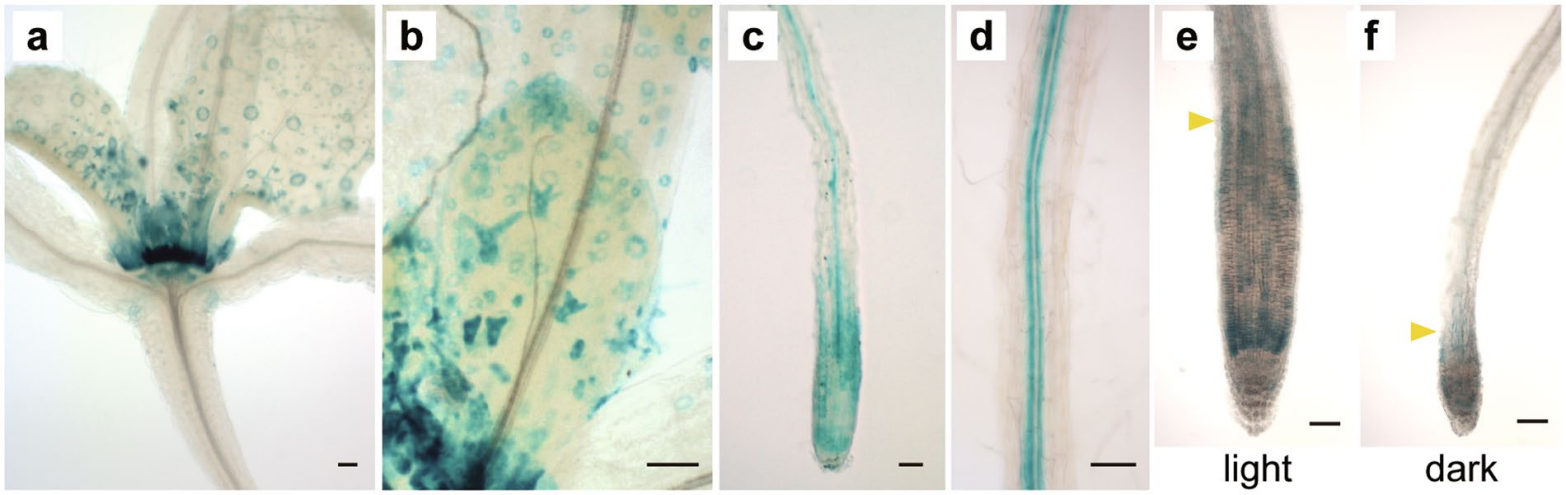
DWF4-GUS expression pattern. (**a**) High expression levels in the shoot apical meristem and surrounding region. (**b**) Expression levels in guard cells and developing trichomes. Expression levels in the root apex and young central cylinder (**c**) as well as the central cylinder (**d**) DWF4-GUS expression levels in roots under light (**e**) and in darkness (**f**) as determined by differential interference contrast microscopy. Arrowheads indicate the borders between the cell division and elongation zones. Scale bars represent 50 µm.

Using the established *DWF4-GUS* plants, we first analysed tissue-specific GUS production. In shoots exposed to light, strong GUS activity was observed in the shoot apical meristem and its periphery (Fig. 2a), as well as in the young trichomes and guard cells of the leaf epidermis (Fig. 2b). In roots, GUS activity was observed in the RAM as well as in the cell division and expansion zones (Fig. 2c) and the central cylinder (Fig. 2d). To examine the possible correlation between root growth and the DWF4-GUS activity level, we compared GUS activity between the roots of *DWF4-GUS* plants grown under light and in darkness. At 7 days after germination (DAG), the GUS activity level was much lower in *DWF4-GUS* plants grown in darkness (Fig. 2f) than in plants exposed to light (Fig. 2e). In darkness, GUS activity in the RAM was observed in the cell division zone, but not in the cell elongation zone (Fig. 2f).

**Figure 3:**
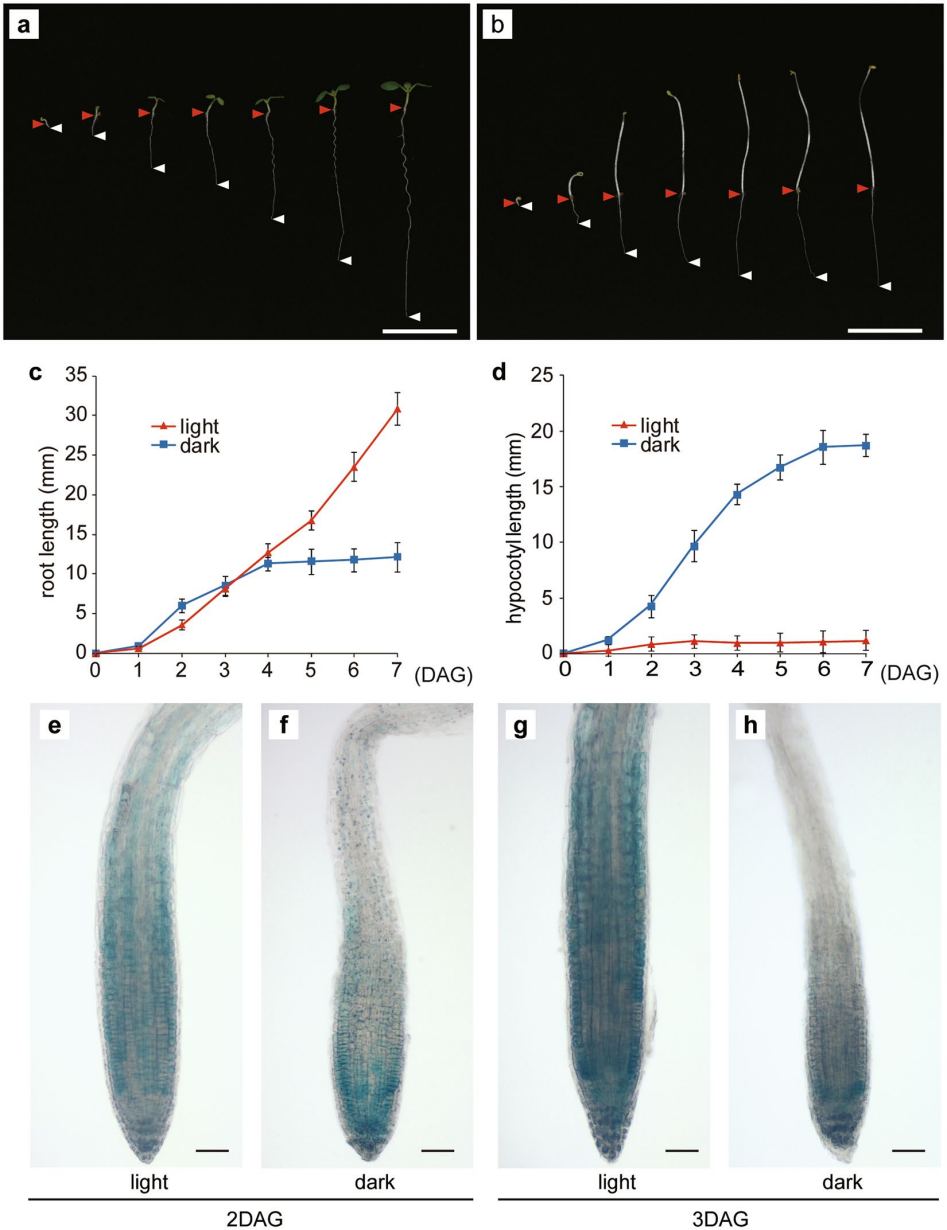
Root and hypocotyl growth and DWF4-GUS expression patterns. Plant growth under light (**a**) and in darkness (**b**) from 1 to 7 days after germination (DAG). Red arrowheads indicate the junctions between the hypocotyl and root, while white arrowheads indicate the root apex positions. Root (**c**) and hypocotyl (**d**) lengths from 1 to 7 DAG [n = 24]. After 3 DAG, the plants grown in darkness exhibited decreased root growth. (**e**–**h**) DWF4-GUS expression pattern in the root meristem. At 2 DAG, there were no differences in DWF4﻿-GUS signals between the root meristems of plants incubated under light (**e**) or in darkness (**f**). At 3 DAG, the DWF4﻿-GUS signal intensity in the roots incubated in darkness (**h**) started to decrease in the meristematic region and cell elongation zone. Scale bars represent 10 mm (**a**,**b**) and 50 µm (**e**–**h**). Error bars represent standard deviations.

We monitored the growth of roots and hypocotyls from 1 to 7 DAG (i.e., during the juvenile stage) in plants incubated in darkness or under light (Fig. 3a–d). The roots of plants grown in darkness were slightly longer than the roots of plants exposed to light until 3 DAG (Fig. 3c). In contrast, hypocotyls grown in darkness were much longer than hypocotyls incubated under light (Fig. 3d). However, after 3 DAG, the roots of plants incubated under light grew faster than the roots maintained in darkness. To reveal the spatio-temporal changes to the DWF4-GUS accumulation pattern, we examined the roots of *DWF4-GUS* plants from 1 to 7 DAG. Analyses of the younger seedlings revealed that GUS activity in the root cell expansion region of dark-grown seedlings did not increase between 2 and 3 DAG (Fig. 3f,h). These results imply that light promotes DWF4 accumulation in root tissues (Fig. 3e,g).

### Light perception in shoots affects root elongation and *DWF4* expression in the root apical meristem

**Figure 4:**
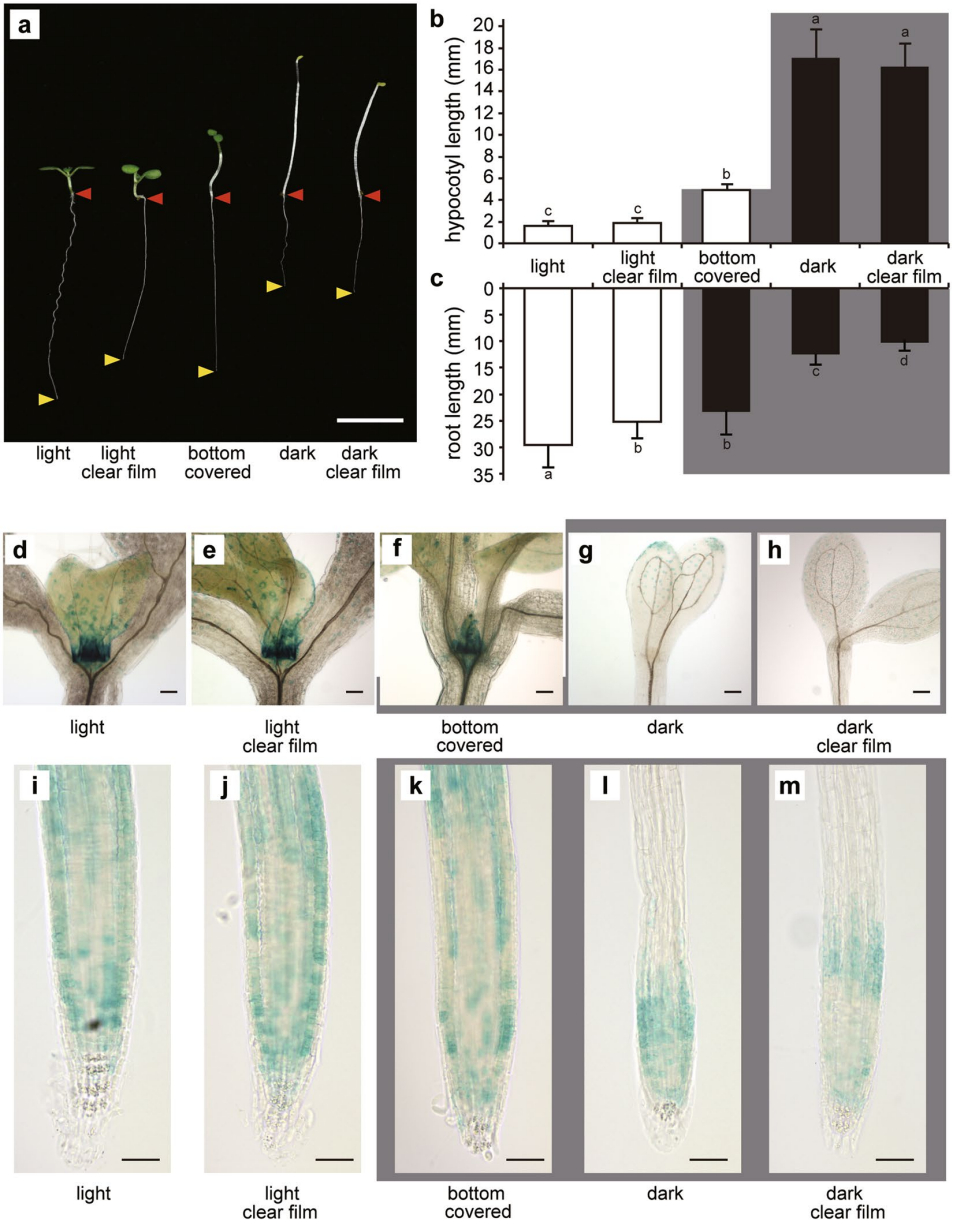
Root elongation and DWF4-GUS expression under light and in darkness according to the bottom half covered growth (BCG) test. The results of the BCG test revealed that light perception in shoots promotes *DWF4* expression in the root apex. (**a**) Root elongation. Red arrowheads indicate the junctions between the hypocotyl and root, while yellow arrowheads indicate the root apex positions. Hypocotyl (**b**) and root (**c**) lengths according to the BCG test. The grey background indicates which part was darkened (n = 32). Means denoted by the same letter are not significantly different (P < 0.05) according to the Tukey–Kramer method. DWF4-GUS expression around the shoot apical meristem (**d**–**h**) and in the root apex (**i**–**m**). Light conditions with no film (**d**,**i**) or with a clear film (**e**,**j**). Shoots under light and roots in darkness covered with a black film (**f**,**k**). Although the root apex was shaded, the GUS signals in the root meristem were stronger than those of plants maintained in darkness. Dark conditions with no film (**g**,**l**) or with a clear film (**h**,**m**). Bars represent 10 mm (**a**) and 50 µm (**d**–**m**). Error bars represent standard deviations.

The results described in the previous section imply that light perception in shoots may induce DWF4 accumulation in the RAM through an unknown systemic mechanism. However, it is possible that the roots were able to perceive light under our experimental conditions. To eliminate this possibility, we developed a “bottom half covered growth (BCG) test”, in which the lower half of plates was covered with black polyethylene film. This resulted in only the shoots being exposed to light, while the roots remained in darkness (Supplementary Fig. S2). Seeds were aseptically placed on Murashige and Skoog (MS) agar medium in the darkened sides of plates, and were then covered with sterilized black film. The plates were positioned vertically and incubated under light until 7 DAG. The seeds were allowed to germinate in darkness under the film. When the elongated hypocotyls became approximately 5 mm long, the shoots were able to perceive light, and the hypocotyls stopped growing (Fig. 4a,b). In the BCG test, there were no significant root length differences between plants grown in darkness and those exposed to light until the shoots started to perceive light (i.e., when hypocotyls were about 5 mm long) as indicated in Fig. 3c,d. The roots began to grow after the plants started to perceive light. At 7 DAG, the seedlings used for the BCG test had much longer roots than the seedlings grown in darkness, and slightly shorter roots than the seedlings grown under light (Fig. 4a,c). To eliminate the possibility that contact effects of the film used to cover the seeds were influencing root growth, we used a clear film to cover control samples in the BCG test (Fig. 4a–c). There were no significant root length differences between the clear film-covered and black film-covered plants in the BCG test. These results suggest that light perception in shoot tissue promotes root growth, even though the roots themselves are maintained in darkness.

We subsequently analysed DWF4-GUS accumulation in plants used for the BCG test (BCG plants). The roots and shoots of BCG plants exhibited high GUS activity levels similar to plants grown under light, but in contrast to plants incubated in darkness (Fig. 4d–m). These results suggest that light perception in shoots promotes DWF4 accumulation in the root apex. Additionally, high DWF4 activity levels in roots may enhance root growth through the activation of cell division and elongation.

**Figure 5:**
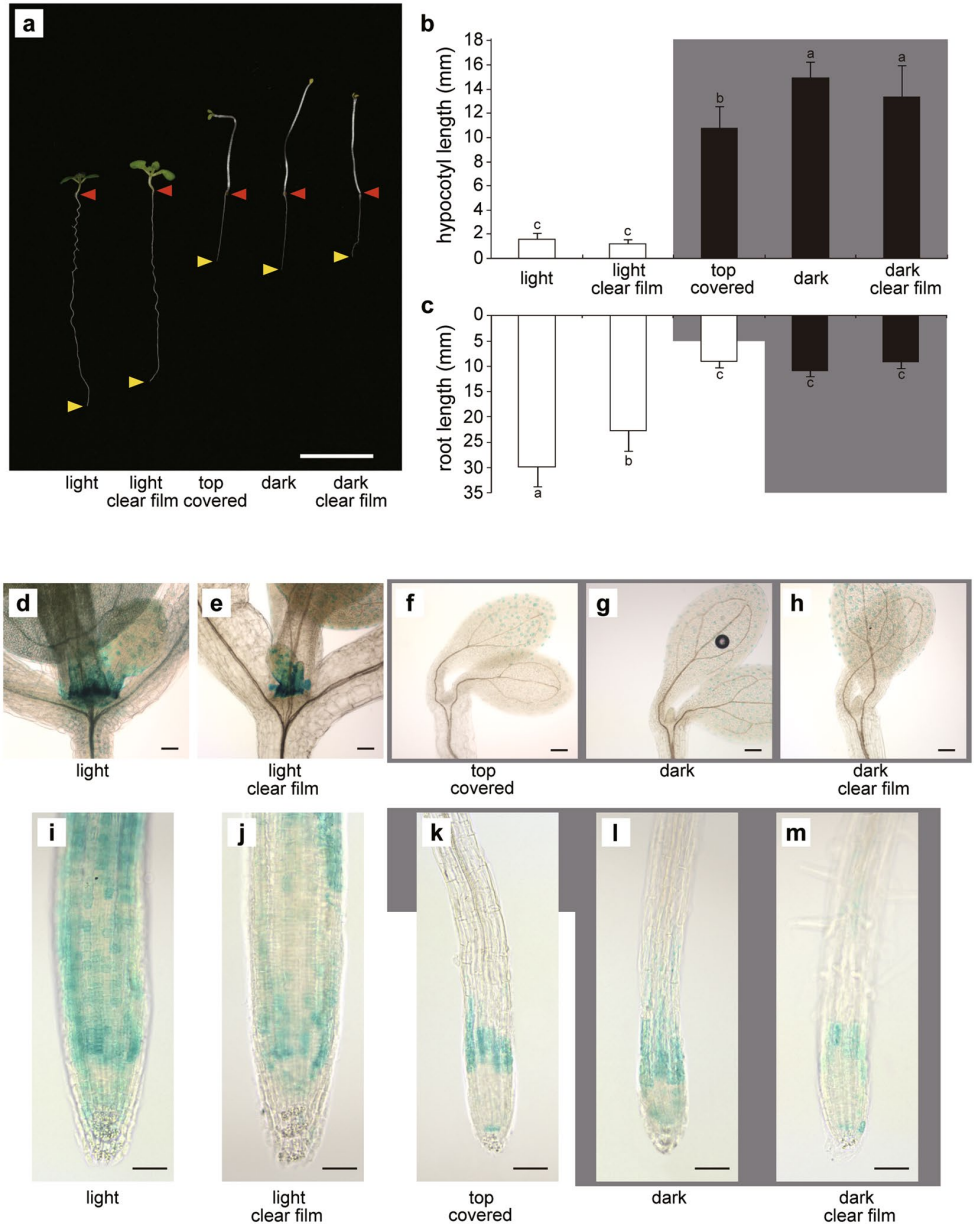
Top half covered growth (TCG) test results revealed that light perception in roots did not promote *DWF4* expression in the root apex. (**a**) Root elongation. Red arrowheads indicate the junctions between the hypocotyl and root, while yellow arrowheads indicate the root apex positions. Hypocotyl (**b**) and root (**c**) lengths according to the TCG test. The grey background indicates which part was darkened [n = 32]. Means denoted by the same letter are not significantly different (P < 0.05) according to the Tukey–Kramer method. DWF4-GUS expression around the shoot apical meristem (**d**–**h**) and in the root apex (**i**–**m**). Light conditions with no film (**d**,**i**) or with a clear film (**e**,**j**). Shoots in darkness covered with a black film and roots under light (**f**,**k**). Although the root apex was exposed to light, the GUS signal in the root meristem was weaker than that of plants that were incubated entirely under light. Dark conditions with no film (**g**,**l**) or with a clear film (**h**,**m**). Bars represent 10 mm (**a**) and 50 µm (**d**–**m**). Error bars represent standard deviations.

To confirm that the light perception in shoots enhances root growth, we devised a “top half covered growth (TCG) test”, in which the shoots are covered with a black film, while the roots are exposed to light (Fig. 5). To avoid phototactic shoot movements, TCG plates were incubated in complete darkness until 3 DAG to promote hypocotyl growth. The plates were then exposed to light. The roots of plants used for the TCG test (TCG roots) were shorter than those of plants covered with clear film, but were almost the same length as the roots of plants grown in darkness (Fig. 5a–c). These results indicate that light perception in roots does not promote root growth. We also analysed DWF4-GUS accumulation in the TCG test (Fig. 5d–m). The GUS activity levels in the root apex of TCG plants (Fig. 5k) were as weak as those of roots incubated in darkness (Fig. 5l,m), suggesting that the perception of light in roots does not promote *DWF4* accumulation in the root apex. We conclude that light perception in shoots, but not in roots, promotes BR biosynthesis in roots, which may enhance root growth.

## Discussion

The BR signalling pathway has been well described, from the effects of light to plant growth (e.g., hypocotyl elongation), but there is relatively little information available regarding the sites where BR is actually synthesized. Our study results indicate that light perception in the aerial tissues affects root growth, possibly through the induction of BR biosynthesis in the root tip regions. Among the enzymes in the BR biosynthesis pathway, DET2, CPD, and DWF4 have been the most extensively studied. The *DWF4* expression level was observed to be lower than that of *CPD* ^40, 50–53^. Consistent with this observation, *DWF4-GUS* plants exhibited localized accumulation patterns in the roots and leaves, which differed from the expression pattern of *proCPD-GUS* reporter plants^11^. Based on these studies, DWF4 is considered the rate-limiting enzyme in the BR biosynthesis pathway^54, 55^. Accordingly, we assumed that the DWF4 accumulation level and pattern significantly affect BR levels during plant growth and development.

We performed qRT-PCR analysis to compare *DWF4* mRNA levels between plants tested in this study (Supplementary Fig. S3). The result showed that root tips under light condition expressed *DWF4* transcript more than those in darkness. But the difference is much smaller than that we observed in DWF4﻿-GUS staining which reflects DWF4 protein accumulation. It is suggested that light might regulate DWF4 protein stability as well.

Wild-type plants grown in darkness produced short roots similar to those of the *dwf4* mutants grown under light or in darkness. This observation suggests that light perception promotes BR biosynthesis in roots, resulting in root elongation. We conducted two experiments to test this hypothesis. First, we completed BCG and TCG tests to reveal the tissue where light is perceived. Our observations strongly indicate that light perception in shoots, but not in roots, is a determining factor for the subsequent root elongation in WT plants, but not in *dwf4* mutants. In general, BR is thought to function as a signal molecule that does not undergo long-distance transport^40, 41^. Second, we established *DWF4-GUS* transgenic lines in a *dwf4-102* background to visualize *DWF4* accumulation in tissues and estimate BR biosynthesis in roots. We observed a tight correlation between light perception in shoots and the DWF4 (DWF4-GUS) accumulation level in root tips. These results imply that some signal, conveyed from the shoots to the roots, induces BR biosynthesis in the root tips, and that the resulting BRs promote root growth *via* cell division and elongation.

We analysed DWF4-GUS accumulation patterns under various conditions, with a particular focus on the meristematic region. We initially confirmed the difference in DWF4-GUS accumulation between light and dark conditions. The GUS signal in the shoot apical meristem and RAM was considerably lower in darkness than under light. We focused on the RAM because the *dwf4-102* mutants exhibited retarded root growth under light and dark conditions. Recent studies have started to investigate BR signalling in *A*. *thaliana* roots^56, 57^. In the RAM of plants grown in darkness, the DWF4﻿-GUS signal was weak and limited to the cell division zone. These results were consistent with the number of meristematic cells and the production of relatively short roots. In terms of the relationship between BRs and root development, the BRs are reportedly indispensable for cell-cycle progression and cell elongation in the RAM^58–62^. Our observations of *DWF4-GUS* plants complement the available tissue-specific information regarding BR biosynthesis in roots developing in darkness. Additionally, our daily observations of *DWF4-GUS* plants for 1 week revealed a decrease in the DWF4﻿-GUS signal in the root apex under dark conditions after 3 DAG (Fig. 3f,h). The root meristem then started to atrophy. We thought that a stable BR supply and DWF4 activity level in the RAM would maintain root meristem activities and the resulting root elongation, especially after 3 DAG. However, BRs promote hypocotyl elongation in darkness. These results suggest that *DWF4* expression is differentially regulated between the hypocotyl and RAM. Therefore, when seeds germinate in darkness, the seedlings may detect their underground position and prioritize hypocotyl elongation over root elongation to perceive light as soon as possible. This would likely increase the survival rate of seedlings during the plant germination stage. We believe these regulatory activities have been established not only for the BR signalling pathway, but also for BR biosynthesis. At this moment, we could not find out what kind of signal(s) carried from above ground tissue to RAM to turn on *DWF4* expression, accumulation and consequent BR biosynthesis.

We introduced a new system, BCG and TCG system, to see plants with shoots and roots under light or dark condition. Xu *et al*. reported an agar-plate method to study root growth^42^. In that method aerial tissues are placed outside the container, where the physical conditions for shoots and roots would be different. Silva-Navas *et al*. reported another system, D-Root, to cultivate plants with roots under different light conditions^43, 44^. The strength of light illumination is more intense than that we used in this analysis. On top of that, our observation was performed with young seedling of less than 7 DAG, while other works were conducted using older plants than ours. When we discuss root growth, we should consider experimental differences and what we would like to compare with natural growth condition.

## Methods

### Plant materials and growth conditions

We used the *A*. *thaliana* Col-0 line as the WT control along with several homozygous and heterozygous *dwf4-102* mutant lines (SALK_020761). Seeds were surface-sterilized with 70% (v/v) ethanol for 2 min, rinsed twice with distilled water, and incubated in 0.05% (v/v) plant preservative mixture (Plant Cell Technology; http://www.plantcelltechnology.com/) for 2 days at 4 °C in darkness. Sterilized seeds were germinated in plates containing half-strength MS basal salt medium supplemented with B5 vitamins, 1.0% (w/v) sucrose, 2 mM 2-morpholinoethanesulfonic acid monohydrate (pH 5.8), and 1.5% (w/v) agarose or in a soil mixture of vermiculite soil and Sakata Supermix-A (Sakata Seed Corporation, http://www.sakataseed.co.jp/). Plants were incubated at 22–23 °C under white light (FHF32EX-N-H, 25 µmol m^−2^ s^−1^; Panasonic, http://panasonic.jp/light). Hypocotyl and root lengths were measured using ImageJ software.

### Plasmid construction

We constructed the pWAT2-pro*DWF4*-*DWF4*-GUS vector to generate *DWF4-GUS*–complemented *dwf4-102* mutant plants. We amplified the following DNA fragments using specific primer sets (Supplementary Table 1): *DWF4* promoter region and the open reading frame (4,936 bp) from *A*. *thaliana* Col-0 genomic DNA, *DWF4* terminator region (561 bp) from *A*. *thaliana* Col-0 genomic DNA, and the GUS gene (1,812 bp) from the pWAT208-MASS-GUS plasmid^63^. All amplified fragments were introduced into the pWAT2 plasmid^63^ to construct the pWAT2-pro *DWF4*-*DWF4*-GUS vector. The primers used to construct this vector are listed in Supplementary Table 1.

### Plant transformation

We introduced pWAT2-pro*DWF4*-*DWF4*-GUS into *Agrobacterium tumefaciens* strain GV3101 cells carrying the pSOUP plasmid^63^. These cells were then used to transform *dwf4-102* heterozygotes using the floral dip method. The *A*. *thaliana* inflorescences were dipped in half-strength MS basal salt medium containing 1.0% (w/v) sucrose, 0.05% (v/v) Silwet L77 solution (pH 5.8), and transgene-carrying *A*. *tumefaciens* cells. The selected plants were then subjected to genotyping using the following primers: Lba1, SALK_020761-RP, and SmaI-*DWF4* Term.-rev (Supplementary Fig. S1a, Supplementary Table S1).

### Histochemical staining of *DWF4-GUS* plants

To analyse DWF4-GUS expression in transgenic plants, samples were fixed in 90% acetone for 15 min on ice and washed twice with 100 mM sodium phosphate buffer (pH 7.0). The samples were then incubated overnight at 37 °C in 100 mM sodium phosphate buffer (pH 7.0), 10 mM sodium EDTA, 5 mM potassium ferrocyanide, 5 mM potassium ferricyanide, 0.1% (v/v) Triton X-100, and 0.5 mg/ml 5-bromo-4-chloro-3-indolyl-β-D-glucuronic acid. The stained samples were rinsed twice with 100 mM sodium phosphate buffer (pH 7.0) and then treated with 70% (v/v) ethanol to deplete chlorophyll. The bleached samples were then hydrated in an ethanol series (70, 30, and 15%). To enhance the size of RAM cells, samples were cleared with chloral hydrate.

### Fluorescence and confocal microscopy

An IX70 epifluorescence microscope (Olympus, http://www.olympus-global.com/en/) equipped with a C7780-20 CCD digital camera (Hamamatsu Photonics, http://www.hamamatsu.com/jp/en/index.html) was used to observe GUS staining. A C^2+^ confocal microscope (Nikon, http://www.nikon.com/index.htm) was used to observe PI staining. Roots harvested from plants at 7 DAG were stained with 2.5 µg/ml PI for 2–3 min and then visualized after an excitation by a Sapphire 561-nm laser. The PI signal was detected with a 552–617-nm bandpass filter. Cell lengths in PI-stained samples were measured using ImageJ software.

### RAM activity analysis

To investigate the activity of cell division and consequent cell elongation in root tips, we followed the method described by Silva-Navas *et al*.^44^. In this study, we assigned the border between meristem and elongating cells, where an adjacent cell had a size longer than by more than 50%.

### Statistical analysis

All statistical analyses were completed using Microsoft Excel 2016 software. Significant differences in multiple comparisons were determined with the Tukey–Kramer method.

## Electronic supplementary material

Supplementary Figures and Table
