# Supplementary material for: Light perception in aerial tissues enhances DWF4 accumulation in root tips and induces root growth
Source: Sci Rep. 2017 May 12;7:1808. doi: 10.1038/s41598-017-01872-4 (PMC5431926; doi:10.1038/s41598-017-01872-4)
Supplement: Supplementary file 1 — Supplementary Figures and Table [file 41598_2017_1872_MOESM1_ESM.pdf]

Supplementary Information for

Light perception in aerial tissues enhances DWF4 accumulation in root tips and induces root growth

J. Sakaguchi and Y. Watanabe

Department of Life Sciences, Graduate School of Arts and Sciences, University of Tokyo

Contents of this file

Figures S1-S3, Tables S1

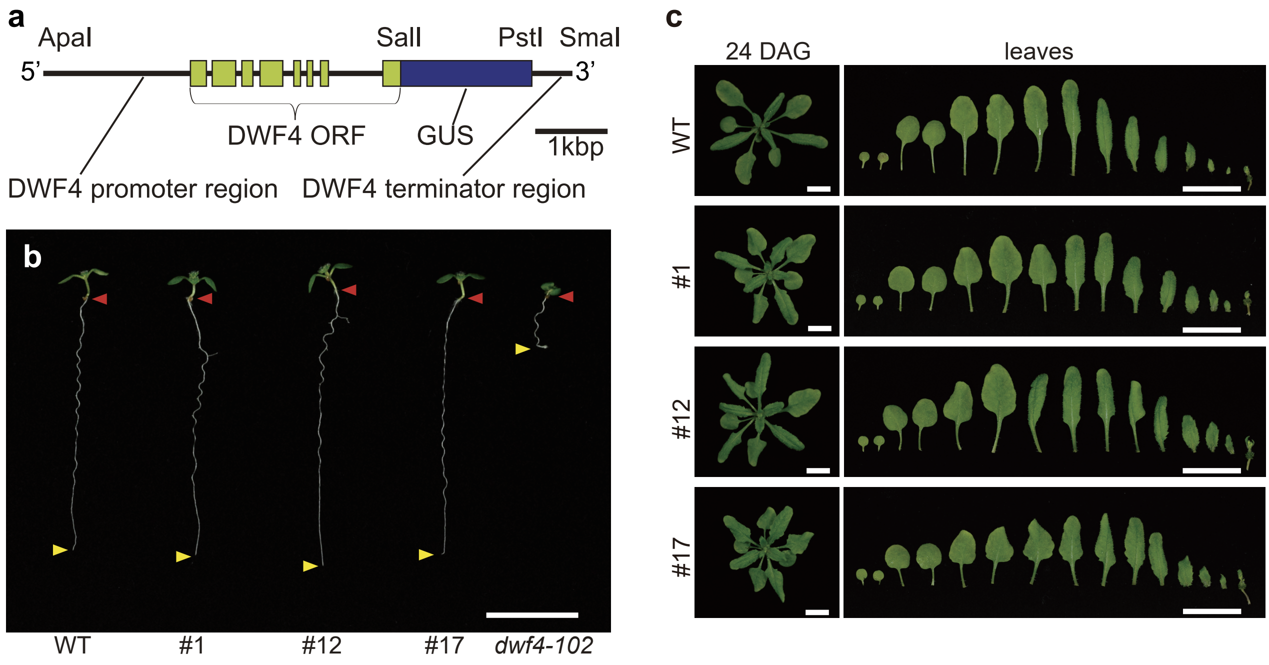

Figure S1. DWF4-GUS complemented the *dwf4* mutant phenotype.

(a) proDWF4-DWF4-GUS-termDWF4 construct.

(b) DWF4-GUS plants at the juvenile stage. *dwf4-102* mutant plants were complemented with the construct presented in (a). Red arrowheads indicate the borders between the hypocotyl and the root, while yellow arrowheads indicate the root apex positions.

(c) DWF4-GUS plants at 24 days after germination. The growth of three independent complementation lines was almost the same as that of wild-type plants. Scale bars represent 10 mm.

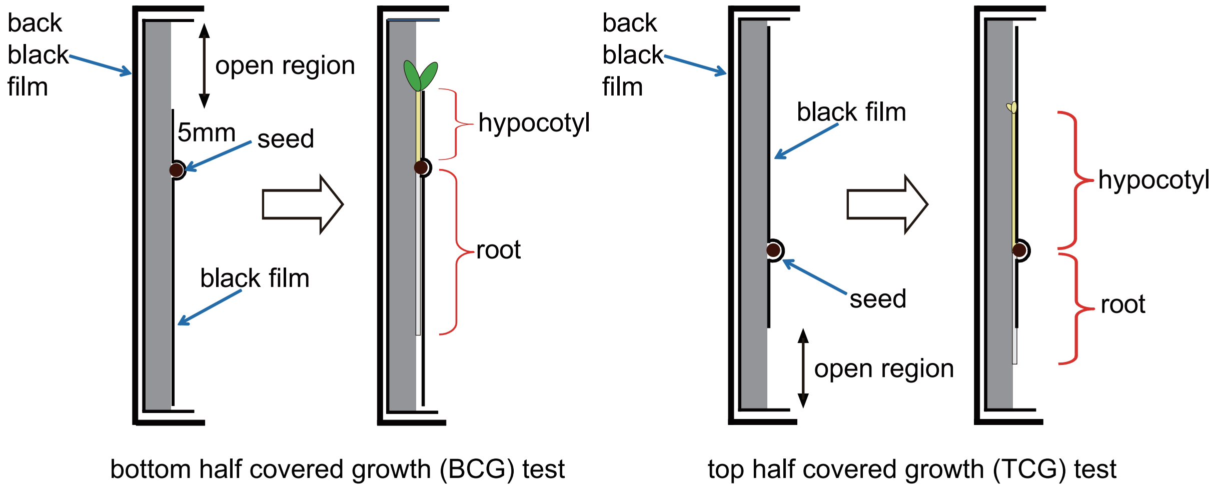

Figure S2. Models of bottom/top covered growth tests.

In BCG growth test, plants germinate under simulated dark condition, and after hypocotyl elongation, shoot start to perceive light but root is kept under the dark. In TCG test, plants germinate under simulated dark condition, and after root elongation, root start to perceive light but shoot is kept under the dark.

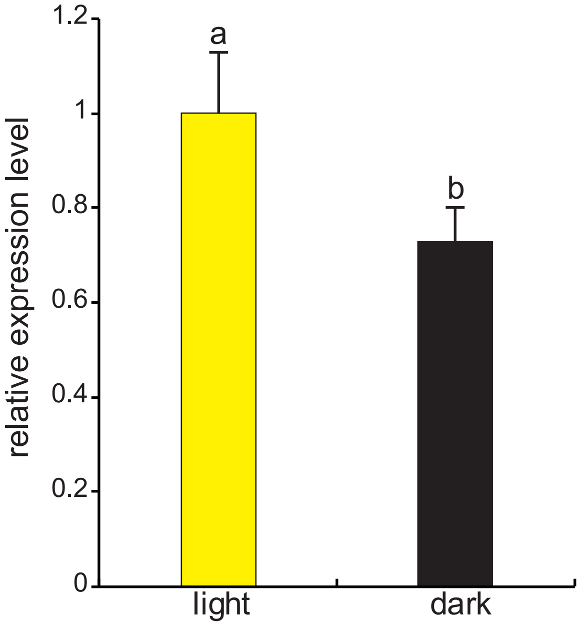

Figure S3. Relative expression levels of *DWF4* mRNA in root tips.

From 7 DAG plants, approx. 3 mm length of root tips were collected and qRT-PCR analysis was carried out. Total RNAs were extracted by NucleoSpin® RNA (MACHEREY-NAGEL, http://www.mn-net.com/).

The first strand cDNA was synthesized by PrimeScript™ RT reagent Kit with gDNA Eraser (Perfect Real Time) (TAKARA-BIO, http://www.takara-bio.co.jp/). One µg of total RNA was added in a 20 µL reaction system and diluted to 10 ng/µL for the following experiments. The KAPA SYBR® FAST qPCR Kit (KAPABIOSYSTEMS, https://www.kapabiosystems.com/) was used for qRT-PCR analyses. All tests were performed in duplicate with a StepOne™ Real-Time PCR Systems (Applied Biosystems, www.thermofisher.com/).

Every PCR was carried out in a 20 µL total volume containing 2 µL first strand cDNA (20 ng total RNA) as template. The relative expression level of each gene was averaged from three replicates using the software installed within equipment using a Quantitation Comparative ΔΔCt method.

Table S1. oligonucleotides used in this study

Oligonucleotide	Sequence
ApaI-proDWF4+DWF4ORF-fwd	AATGGGCCCTAAACAAATATGTTAATCAATTAGTGGTC
SalI-proDWF4+DWF4ORF-rev	GACGTCGACCAGAATACGAGAAACCCTAATAGGCAAACC
PstI-DWF4Term.-fwd	AAACTGCAGAAAAAAAAAAAGATGAAAGTATTTTTATTC
SmaI-DWF4Term.-rev	TCCCGGGTGAGAGCTATTGTTATTCATTTAGTCCTTTTG
SalI-GUS-fwd	TTAGTCGACATGTTACGTCCTGTAGAAACCCCAACC
PstI-GUS-rev	ACTCTGCAGTCATTGTTTGCCTCCCTGCTGC
SALK_020761-RP	CCGGACATGAGACTTCTTCTG
SALK_020761-LP	GGCAGCTCCTACGTCATTAAAG
LBa1	TGGTTCACGTAGTGGGCCATCG
qRT-PCR_RPL7D-fwd	CCGGGCTAAACAGTACTCCA
qRT-PCR_RPL7D-rev	TTCAGCTGGATTAATTCCCTTT
qRT-PCR_DWF4_5'-fwd	CAACATGTCTCCAAGTATGG
qRT-PCR_DWF4_5'-rev	GACGTGCGTGACTTAAGAAG
